# Transplantation of induced pluripotent stem cell-derived renal stem cells improved acute kidney injury

**DOI:** 10.1186/s13578-015-0040-z

**Published:** 2015-08-20

**Authors:** Qing Li, Shou-fu Tian, Ye Guo, Xin Niu, Bin Hu, Shang-chun Guo, Nian-song Wang, Yang Wang

**Affiliations:** Institute of Microsurgery on Extremities, Shanghai Jiao Tong University Affiliated Sixth People’s Hospital, Shanghai, 200233 China; Department Nephrology and Rheumatology, Shanghai Jiao Tong University Affiliated Sixth People’s Hospital, Shanghai, 200233 China; Department of Integration of Traditional Chinese and Western Medicine, The First Affiliated Hospital of Soochow University, Suzhou, 215006 China; Department of Neurosurgery, Shandong Jining No. 1 People’s Hospital, Jining, 272011 China

**Keywords:** Acute kidney injury, iPSC-derived RPCs, Renal ischemia–reperfusion injury, Hydrogel

## Abstract

**Background:**

Acute kidney injury (AKI) is a severe disease with high morbidity and mortality. Methods that promote repair of the injured kidney have been extensively investigated. Cell-based therapy with mesenchymal stem cells or renal progenitor cells (RPCs) resident in the kidney has appeared to be an effective strategy for the treatment of AKI. Embryonic stem cells or induced pluripotent stem cells (iPSCs) are also utilized for AKI recovery. However, the therapeutic effect of iPSC-derived RPCs for AKI has yet to be determined.

**Methods:**

In this study, we induced iPSCs differentiation into RPCs using a nephrogenic cocktail of factors combined with the renal epithelial cell growth medium. We then established the rat ischemia–reperfusion injury (IR) model and transplanted the iPSC-derived RPCs into the injured rats in combination with the hydrogel. Next, we examined the renal function-related markers and renal histology to assess the therapeutic effect of the injected cells. Moreover, we investigated the mechanism by which iPSC-derived RPCs affect AKI caused by IR.

**Results:**

We showed that the differentiation efficiency of iPSCs to RPCs increased when cultured with renal epithelial cell growth medium after stimulation with a nephrogenic cocktail of factors. The transplantation of iPSC-derived RPCs decreased the levels of biomarkers indicative of renal injury and attenuated the necrosis and apoptosis of renal tissues, but resulted in the up-regulation of renal tubules formation, cell proliferation, and the expression of pro-renal factors.

**Conclusion:**

Our results revealed that iPSC-derived RPCs can protect AKI rat from renal function impairment and severe tubular injury by up-regulating the renal tubules formation, promoting cell proliferation, reducing apoptosis, and regulating the microenvironment in the injured kidney.

**Electronic supplementary material:**

The online version of this article (doi:10.1186/s13578-015-0040-z) contains supplementary material, which is available to authorized users.

## Background

Acute kidney injury (AKI) is characterized by acute tubular injury and a rapid decline in renal function [1]. Renal ischemia is one of the most common causes of AKI. AKI has recently received increasing attention due to the high mortality and morbidity rates of the syndrome [2]. Although pharmacological therapy, modern dialysis techniques, and intermittent or continuous renal replacement therapies exist, the therapeutic approaches for AKI remain very limited. Thus, the establishment of novel therapeutic strategies for ischemic AKI is urgently needed.

In response to acute injury, the adult kidney shows some level of regeneration characterized by the proliferation of the surviving cells and the replacement of the necrotic tubular cells with functional tubular epithelium [3]. The process is hypothesized to involve epithelial cell dedifferentiation, interstitial cell transdifferentiation and/or activation of quiescent stem cells. Over the last few years, renal progenitor cells (RPCs) have been isolated from the kidney [4–7]. Several studies have indicated that RPCs appear to play important roles in kidney repair under various pathological conditions [4, 8–10]. However, progenitor cells in the adult kidney are rare, which limits the application of these cells. Therefore, we resorted to other types of stem cells.

Stem cells, including embryonic stem cells (ESCs) and adult stem cells, have the potential of self-renewal and differentiation into multiple cell types and therefore hold great promise in regenerative medicine [11–13]. It was recently reported that stem cells exhibit therapeutic potential for AKI [14–17]. Several studies have demonstrated that the exposure of ESCs to factors required for renal specification, such as retinoic acid, activin A and bone morphogenic proteins (BMPs), does induce their differentiation into renal lineage cells in vitro [18–20]. Vigneau et al. [10] showed that the transplantation of mouse ESCs-derived RPCs can result in the stable integration into proximal tubules with normal morphology and normal polarization injection into developing live newborn mouse kidneys, suggesting the potential of ESCs for application in regenerative therapies. However, the use of ESCs for human therapy raises several safety and ethical concerns.

Induced pluripotent stem cells (iPSCs) are a type of newly defined stem cell with properties similar to those of ESCs in terms of self-renewal and differentiation. These cells were first generated by Yamanaka and colleagues [21]. Since their discovery, iPSCs have shown their advantages in regenerative medicine. iPSCs have also been reported to be effective for AKI. Lee and colleagues [16] showed that the injection of iPSCs into the kidney improves renal function and attenuates renal tubular injury in AKI caused by ischemia–reperfusion injury (IR) and eventually improves the survival rate of rats with AKI. Although iPSCs are an alternative potential cell source for stem cell therapy to protect against AKI, there is also a risk of tumor formation. Therefore, we investigated whether iPSC-derived RPCs have a therapeutic effect on AKI.

In the present study, we examined whether iPSC-derived RPCs can protect against AKI in a rate model of IR. We showed that the differentiation efficiency of iPSCs to RPCs increased when cultured with Renal Epithelial Cell Growth Medium after stimulation with a nephrogenic cocktail of factors. The injection of iPSC-derived RPCs combined with hydrogel, which provides a 3-D structure to the cells, into the kidney of rats with IR could improve renal function and attenuate tubular injury by forming renal tubules in the recipient rat kidney directly, promoting cell proliferation, reducing apoptosis, and regulating the microenvironment in the injured kidney.

## Results

### Efficient differentiation of RPCs from mouse iPSCs

**Fig. 1:**
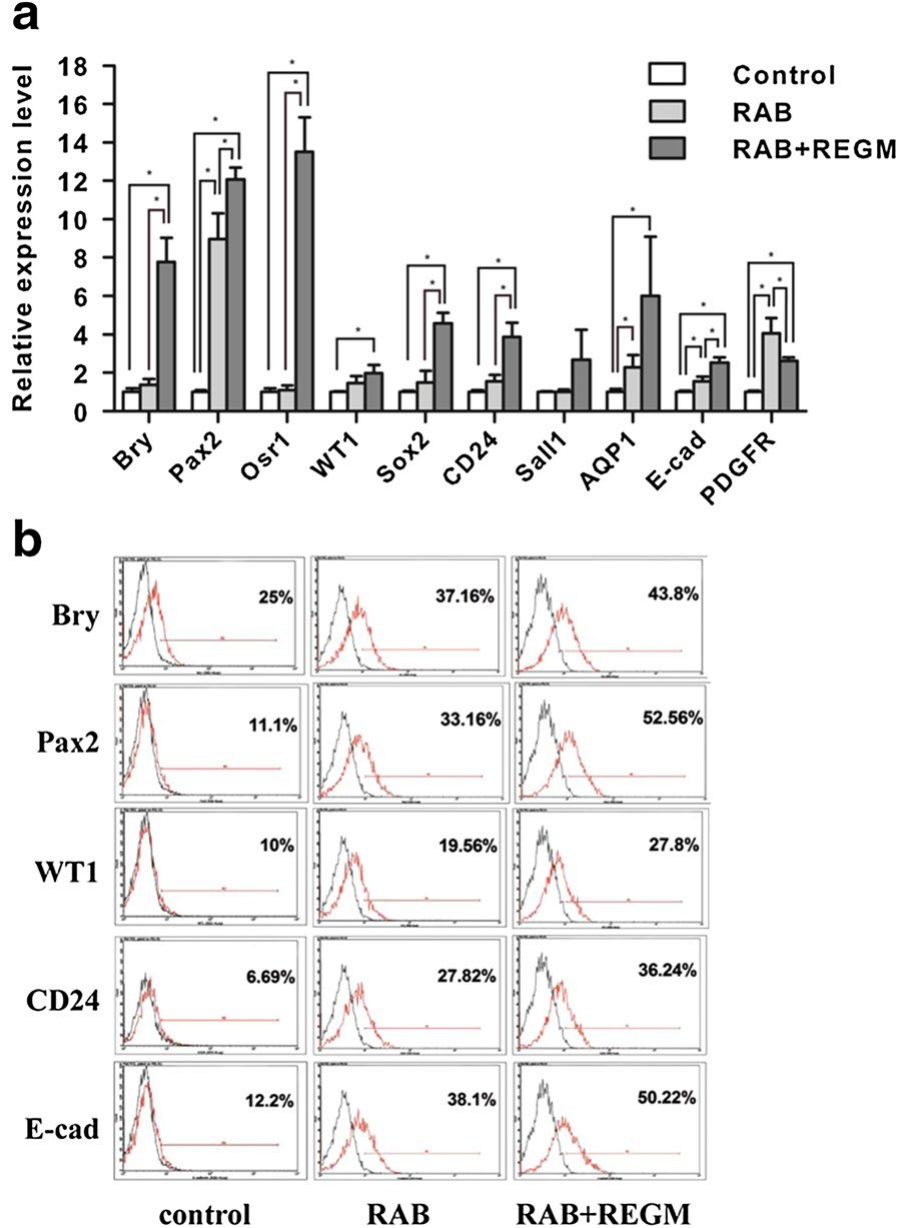
Efficient differentiation of renal progenitor cells from mouse induced pluripotent stem cells (iPSCs). The renal differentiation of mouse iPSCs was initiated by the formation of embryoid bodies (EBs). After 2 days, retinoic acid, activin A, and BMP7 were added to the differentiation medium, and the cells were exposed to this supplemented media for 5 days (RAB group). After incubation with growth factors for 5 days, the EBs in the RAB + REGM group were cultured in renal epithelial cell growth medium for another 5 days. **a** Real-time PCR analysis of the expression of mesoderm (Bry) and renal lineage genes (Pax2, Osr1, WT1, Six2, CD24, Sal1, AQP1, E-cad and PDGFR). **b** Flow cytometry analysis of the expression of mesoderm renal progenitor-related genes. *Black line*, cells stained with isotype control; *Red line*, cells stained with indicated antibodies. *P < 0.05

To investigate whether iPSC-derived RPCs can improve AKI, we first used a nephrogenic cocktail of factors (including retinoic acid, activin A, and BMP7) to induce iPSCs differentiation into RPCs (Additional file 1: Figure S1). The iPS cell line used in this study is tagged with a ubiquitously expressed *gfp* gene. The real-time PCR analysis showed that the expression of the RPC markers PAX2, WT1, Six2 and CD24 increased after RAB treatment (Fig. 1a), indicating that RPCs were successfully induced. As shown by flow cytometry analysis, the PAX2, WT1 and CD24 positive cells were 33.16, 19.56 and 27.82 % respectively (Fig. 1b). Additionally, the mature renal markers AQP1 and E-cadherin were up-regulated upon RAB stimulation (Fig. 1a). To improve the differentiation efficiency of iPSCs into RPCs, the cells were cultured in renal epithelial cell growth medium for another 5 days (Additional file 1: Figure S1). As expected, the expression of the RPCs markers was further up-regulated compared with that of the RAB group, as shown by real-time PCR analysis after 5 days of differentiation in RAB medium (Fig. 1a). Moreover, the PAX2, WT1, and CD24 single-positive cells increased to 52.56, 27.8 and 36.24 % respectively (Fig. 1b), indicating that more RPCs were generated in the RAB + REGM group.

### Therapeutic effect of iPSC-derived RPCs in rats with IR-induced AKI

**Fig. 2:**
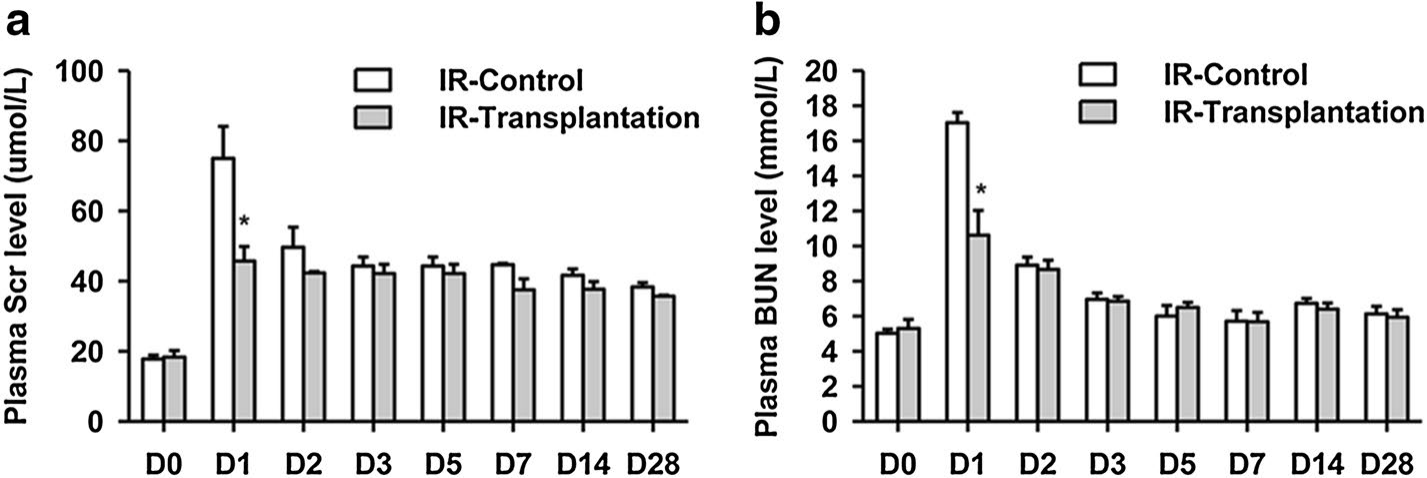
Generation of rat renal ischemia–reperfusion model. The rat renal ischemia–reperfusion injury (IR) model was generated by occlusion of the left renal artery followed by reperfusion and right kidney removal. iPSC-derived renal progenitor cells were injected into the renal parenchyma of the injured rats in combination with a hydrogel. The Scr (**a**) and BUN (**b**) levels in the plasma were detected at the indicated times. *P < 0.05 vs. IR-Control

**Fig. 3:**
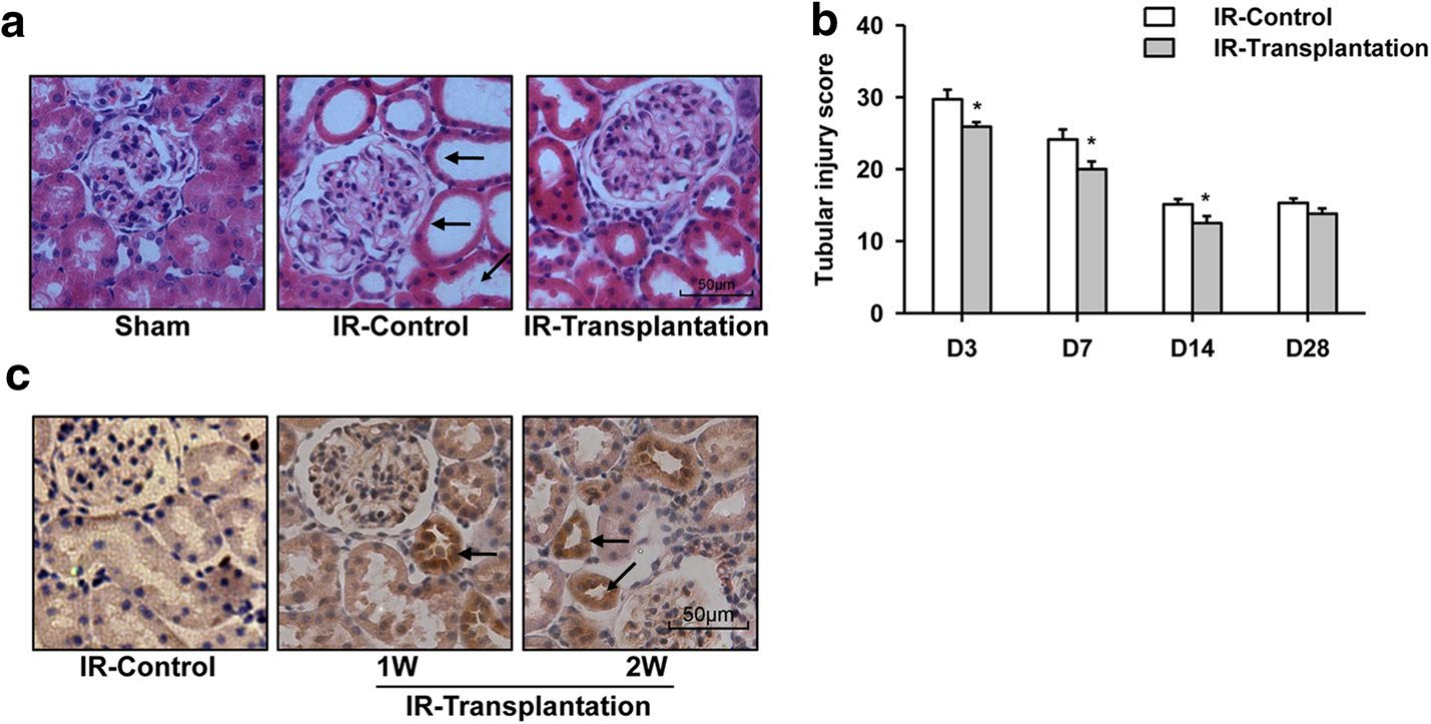
Transplantation of iPSC-derived renal progenitor cells improved renal tubular damage caused by ischemia–reperfusion. **a** Pathological changes in the kidney on the third day after surgery, as shown by H&E staining. Luminal debris, loss of brush borders and lumen expansion are observed in the injured groups (*black arrow*). **b** The transplantation of iPSC-derived renal progenitor cells reduced the acute tubular necrosis scores. **c** The immunohistochemistry analysis indicated that the injected GFP-positive cells formed a tubular structure (*black arrow*) in the recipient organ at the indicated times after transplantation. *P < 0.05 vs. IR-Control. *Scale bar* 50 μm

Next, the rat IR model was generated by occlusion of the left renal artery followed by reperfusion and right kidney removal. iPSC-derived RPCs from the RAB + REGM group were injected directly into the renal parenchyma of the injured rats in combination with the hydrogel. The control group (IR-control) was injected with PBS combined with the hydrogel. The levels of BUN and Scr were detected at the indicated times. The plasma levels of BUN and Scr reached their peak levels at day 1 of renal IR in all groups and then decreased gradually (Fig. 2a, b). After RPC transplantation, the plasma levels of BUN and Scr were lower than those of the IR-control group (Fig. 2a, b). To test whether the RPC transplantation has beneficial effects on regeneration after IR, the rats were sacrificed on days 3, 7, 14, and 28, and the renal histology was examined. H&E staining showed that the proximal tubule in the renal cortex in the control group exhibited acute injury and necrosis 3 days after surgery (Fig. 3a): the brush border of some epithelial cells disappeared; the lumen expanded; some of the tubular epithelial cells detached from the basement membrane; the tubule was obstructed with cellular debris; and the interstitial tubule was infiltrated with inflammatory cells. The histological features of necrotic injury were still severe 7 days after ischemia and began to improve on days 14 and 28. The injury phenotype was decreased in the rat kidney as a result of RPC transplantation. The quantitative analysis of the renal tubular necrosis using the grading scores of Paller et al. [22] is shown in Fig. 3b. These results suggest that the transplantation of iPSC-derived RPCs could improve renal regeneration and function after AKI.

### Mechanistic study of the improvement in the renal tubular damage caused by IR as a result of iPSC-derived RPC transplantation

**Fig. 4:**
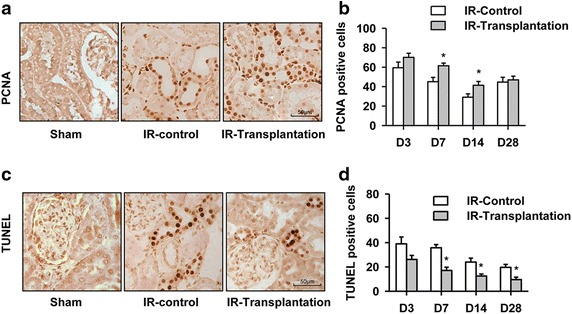
Cell proliferation was increased and apoptosis was decreased in the transplanted renal parenchyma. **a** The proliferation of renal tubular cells was increased 7 days after cell transplantation, as determined by PCNA staining. **b** Quantitative analysis of PCNA-positive cells in **a**. **c** Renal tubular cell apoptosis was decreased 7 days after cell transplantation, as determined by TUNEL assay. **d** Quantitative analysis of **c**. *P < 0.05 vs. IR-Control. *Scale bar* 50 μm

**Fig. 5:**
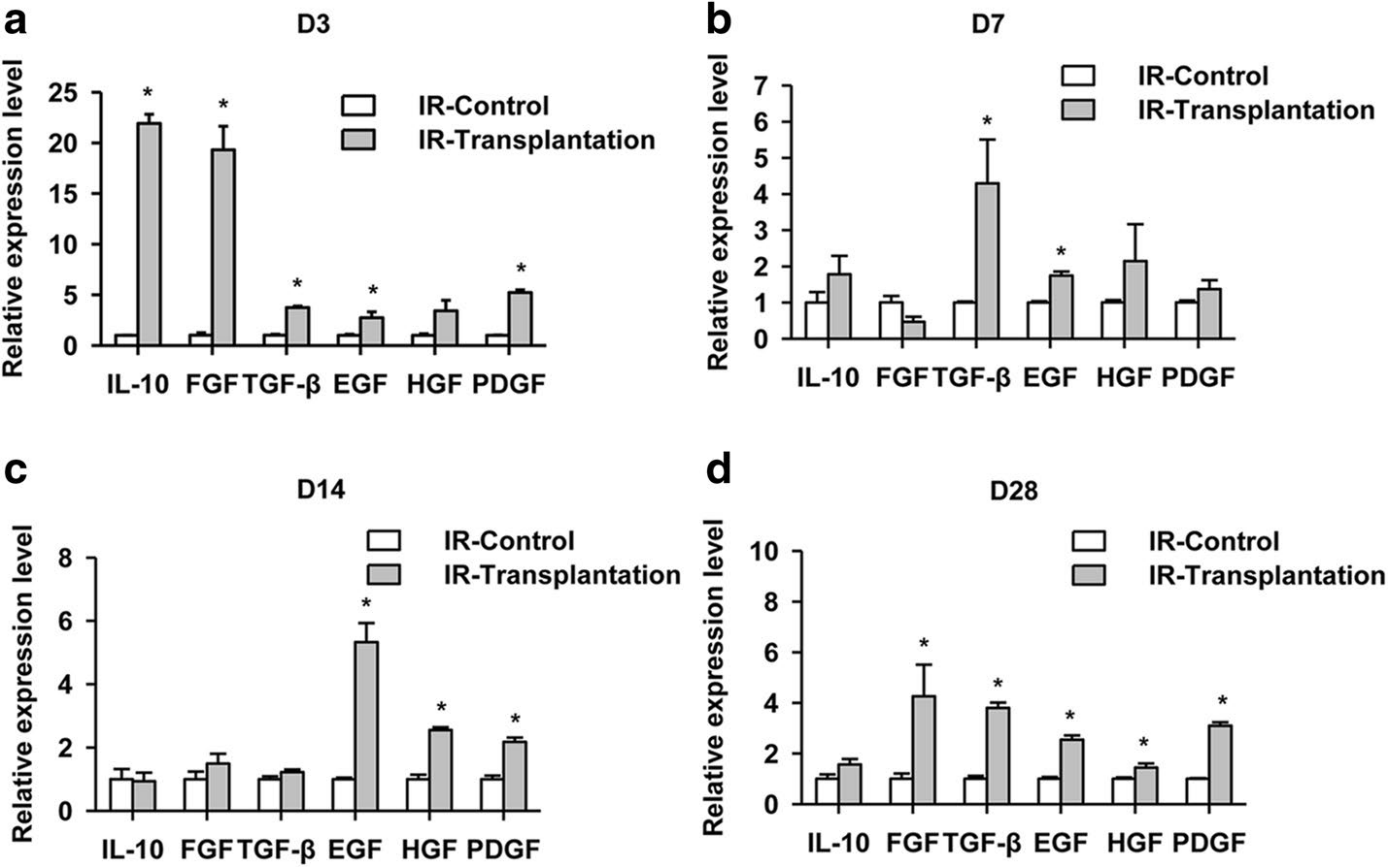
Anti-inflammatory factors and pro-renal tubular repair factors increased after renal progenitor cell transplantation. Real-time PCR analysis of the expression of anti-inflammatory factors (IL-10, FGF, and TGF-β) and pro-renal tubular repair factors (EGF, HGF, and PDGF) on D3, D7, D14, and D28. The anti-inflammatory factors and pro-renal tubular repair factors in the renal cortex of rats after cell transplantation are up-regulated compared with the levels observed in the control group. *P < 0.05 vs. IR-Control

We further investigated the mechanism through which RPCs relieve acute renal injury caused by IR. Previous studies have reported that mouse kidney progenitor cells accelerate renal regeneration after ischemic injury by differentiation into epithelial cells and incorporating into the renal tubule [9, 23–25]. Thus, we first examined the localization of the transplanted iPSC-derived RPCs. Because the iPS cell line used in our study carries a ubiquitously expressed *gfp* gene, it is convenient to trace the injected cells. Using immunohistochemistry, we showed that GFP-positive cells began to form a tubular structure in the recipient kidney 7 days after injection (Fig. 3c). Importantly, no tumor was detected even 3 months after transplantation. Next, the effects of the RPCs on the proliferation and apoptosis of cells in the injured kidney were examined by PCNA staining and TUNEL analyses, respectively. The results showed that the number of PCNA-positive cells increased in the transplanted group compared with that found in the control group on days 7 after injection (Fig. 4a, b), indicating the presence of a greater number of proliferating renal tubular epithelial cells in the kidney after RPC transplantation. In contrast, the cell apoptosis on days 7 was reduced in the RPC-transplanted group, as shown by TUNEL analysis (Fig. 4c, d). Furthermore, we detected the expression levels of the anti-inflammatory factors interleukin-10 (IL-10), basic fibroblast growth factor (bFGF), and transforming growth factor β1 (TGF-β1) and growth factors promoting renal tubular cell repair, namely epidermal growth factor (EGF), hepatocyte growth factor (HGF), and platelet-derived growth factor (PDGF). The real-time PCR analysis showed that the expression of these genes was upregulated to different extents after RPC transplantation (Fig. 5). Altogether, these data indicate that iPSC-derived RPC transplantation improves renal function after IR by directly incorporating into the renal tubule, promoting cell proliferation, reducing apoptosis, and upregulating the expression of pro-renal factors.

## Discussion

AKI is an emerging worldwide public health problem with high mortality and morbidity rates and has thus attracted increasing attention. Although traditional therapies show benefits in the treatment of AKI, the therapeutic approaches are still very limited. Recently, stem cell-based therapy appeared to be a potential strategy for the prevention of AKI [26]. In the present study, we provide the first demonstration that the transplantation of iPSC-derived RPCs improves renal regeneration and function in a rat model of IR-induced AKI, and the protective effect of the RPCs was mediated by their direct differentiation into renal lineage cells and incorporation into the renal tubule, which resulted in the promotion of cell proliferation and the reduction of apoptosis in the damaged zone. Furthermore, the transplantation of iPSC-derived RPCs promoted the expression of anti-inflammatory factors and pro-renal tubular repair factors, suggesting that RPCs can regulate the microenvironment and facilitate cell survival in the injured kidney.

After ischemic injury, RPCs and mesenchymal stem cells (MSCs) resident in the kidney are activated and show some level of regenerative ability. Several studies have shown the therapeutic potential of isolated primary RPCs or MSCs in experimental AKI models. However, these cells are not available in large volumes. Recent advances demonstrated that iPSCs may be more readily accessible for kidney regeneration [16]. Therefore, in our study, iPSCs were differentiated into renal lineage cells to obtain a sufficient number of RPCs. To the traditional induction medium, we added renal specific growth medium for further induction. Using this modified protocol, the differentiation efficiency of iPSCs into RPCs and mature renal epithelial cells increased. Because kidney regeneration requires many functionally distinct cell types of the nephron, the transplantation of all renal cell types derived from iPSCs may offer the advantage of generating more than one cell type of the adult kidney.

In previous studies, stem cells for the treatment of AKI were mainly injected through the tail vein or the intrarenal artery [9, 16], and the transplanted stem cells in the circulation are mobilized to the injury site in the kidney mediated by chemokines/cytokines and receptor interaction and thereafter enhance renal regeneration. However, the homing efficiency cannot be determined. In our study, RPCs were injected directly into the renal parenchyma in combination with a hydrogel. A hydrogel consists of a network of polymer chains, and the function and structure of a hydrogel are similar to those of a natural extracellular matrix. Therefore, hydrogels are currently widely used as scaffolds in tissue engineering [27–30]. The hydrogel used in our work is composed of polypeptides and can be degraded into natural amino acids in vivo; thus, the hydrogel has no hazardous effect on the injured tissue. After being injected into the renal parenchyma with the RPCs, the liquid hydrogel will quickly transform into a solid structure, which provides a three-dimensional microenvironment for RPC adhesion, migration, proliferation and differentiation. Our results showed that the exogenous cells formed a tubular structure in the injured kidney, indicating that the RPCs could migrate out of the hydrogel and participate in renal regeneration.

The therapeutic mechanisms of primary RPCs for AKI were recently investigated in several studies [9, 23, 31]. It has been reported that the transplanted RPCs can differentiate into renal tubule cells and vessel endothelial cells with the expression of E-cadherin and CD34 [23] and therefore accelerate renal regeneration and prolong survival after AKI. Our result that iPSC-derived RPCs can differentiate into renal tubule cells and form renal tubules in the injured kidney further supports this hypothesis. Moreover, we showed that the transplantation of iPSC-derived RPCs promoted cell proliferation and decreased apoptotic events. The cells also exhibited an anti-inflammatory effect by increasing the expression of anti-inflammatory factors, including TGF-β1. A recent study [32] reported that the presence of TGF-β1 in the homogenate of acute IR-injured renal tissue markedly increases the CXCR4 surface expression of MSCs and promotes the migration of MSCs to SDF-1. Therefore, we hypothesized that the transplantation of RPCs increased the level of TGF-β1 in the injured zone, which in turn regulated the CXCR4 expression of the RPCs, contributing to the migration of the RPCs out of the hydrogel to the injured sites. However, this proposed mechanism requires further investigation.

Collectively, the present study demonstrated that the transplantation of iPSC-derived RPCs is able to protect against kidney injury in an experimental IR rat model. These cells function in both differentiation-dependent and -independent manners by directly forming renal tubules and exerting pro-proliferative, anti-apoptotic, and anti-inflammatory effects. Our data suggest that iPSC-derived RPCs are a potential cellular resource for stem cell-based therapy for IR-induced AKI.

## Conclusions

This study reports a modified method for the differentiation of iPSCs into RPCs and provides experimental evidence that iPSC-derived RPCs combined with a hydrogel can improve renal function in AKI and reduce tubular injury in an animal IR model. We believe that iPSC-derived RPCs hold great potential to be used as seed cells for personalized treatment in regenerative medicine.

## Methods

### IPSCs culture

Undifferentiated iPSCs were routinely cultured and expanded on mitotically inactivated MEFs (50,000 cells/cm^2^) in six-well culture plates with mES medium: DMEM supplemented with 15 % FBS, l-glutamine, nonessential amino acids, β-mercaptoethanol (Gibco), and 1000 U/ml LIF (Millipore). The iPS cell line used in the present study was purchased from SiDanSai Biotechnology (Shanghai, China). The sequence of *gfp* gene, for tracing the iPSCs and iPSC-derived cells, was inserted into the chromosome of iPSCs. iPSCs transduced with this sequence showed robust expression of GFP.

### Renal differentiation of murine iPSCs

iPSCs were differentiated through embryoid body (EB) formation in EB medium (DMEM supplemented with 15 % FBS, l-glutamine, nonessential amino acids, and β-mercaptoethanol). On day 3 of differentiation, the EBs were transferred to gelatin-coated culture dishes at a density of 10 EBs per 1 ml of medium. The cells in the control group were maintained in EB medium for 5 days, and the medium was changed every 2 days. The cells in the induction group were exposed to a nephrogenic cocktail of factors, including 0.1 μM RA (Sigma), 10 ng/ml activin-A (PeproTech), and 50 ng/ml BMP7 (PeproTech), in the EB medium for 5 days. The induced cells were then cultured in the EB medium alone (RAB group) or in Renal Epithelial Cell Growth Medium (RAB + REGM group) for another 5 days and harvested for further analyses.

### Quantitative reverse-transcriptase polymerase chain reaction (RT-PCR)

The total RNA from cells or tissues was extracted using the TRIzol reagent according to the protocol delineated by the manufacturer (Invitrogen-Life Technologies). One microgram of total RNA was reverse transcribed into cDNA with oligo-dT primers and reverse transcriptase (Invitrogen-Life Technologies). Real-time quantitative PCR was performed using SYBR Green (Toyobo Co, Japan). The primers used in the amplification reaction are shown in Table 1.

**Table 1:** Primers used for quantitative reverse-transcriptase polymerase chain reaction (RT-PCR)

Genes	Forward primer (5′–3′)	Reverse primer (5′–3′)
m-PAX2	AAGCCCGGAGTGATTGGTG	CAGGCGAACATAGTCGGGTT
m-WT1	CAGTTCCCCAACCATTCCTT	AAGCTGGGAGGTCATTTGGT
m-Brachyury (Bry)	ACCCAGACTCGCCCAATTTT	GGAAAGCAGTGGCTGGTGAT
m-CD24	GTTGCACCGTTTCCCGGTAA	CCCCTCTGGTGGTAGCGTTA
m-Osr1	ATTCACCCTAAGCCAGAGATCA	AGGCCAGGTTAGCGAAATCAA
m-Six2	CTCACCACCACGCAAGTCAG	CTCGGAACTGCCTAGCACC
m-Sall1	CTCAACATTTCCAATCCGACCC	GGCATCCTTGCTCTTAGTGGG
m-E-cad	CTCCAGTCATAGGGAGCTGTC	TCTTCTGAGACCTGGGTACAC
m-AQP1	AGGCTTCAATTACCCACTGGA	GTGAGCACCGCTGATGTGA
m-PDGFR	CCACACCCTTGGGGAATAGT	TTTGAAGGGCAAGGAATGTG
m-GAPDH	ACTTCAACAGCAACTCCCACTC	TAGGCCCCTCCTGTTATTATGG
r-IL-10	TAACTGCACCCACTTCCCAGT	TGGCAACCCAAGTAACCCTTA
r-bFGF	CGACCCACACGTCAAACTAC	CCAGGCGTTCAAAGAAGAAA
r-TGF-β1	GCCCTGGATACCAACTACTGCT	GTTGGTTGTAGAGGGCAAGGAC
r-EGF	CGTCTCAGTGGTCATGGATTT	TCAGAAGAACACGGGAATTGT
r-HGF	CTCTCGTTCCTTGGGATTATTG	GATCCCCCACAGATGTGTTTAT
r-PDGF	ATCGAGCCAAGACACCTCAAAC	ATGGGGGCAGTACAGCAAATAC
r-GAPDH	AGTGCCAGCCTCGTCTCATAG	CGTTGAACTTGCCGTGGGTAG

### Flow cytometry

The differentiated cells were detached from the flask using accutase (Invitrogen-Life Technologies). The single cells were fixed with 4 % paraformaldehyde (Sigma) for 15 min, permeabilized with 0.5 % Triton x-100 (Sigma), and blocked with 3 % BSA (Gibco) for 30 min at room temperature. The cells were then incubated with the following primary antibodies for 1 h: Pax2 (1:100; Abcam), Bry (1:100; Abcam), E-cadherin (1:100; Cell Signaling Technology), CD24 (1:200; BD Biosciences), and WT1 (1:50; Santa Cruz Biotechnology Inc). After washing three times, the cells were incubated with the secondary antibody conjugated to Alexa Fluor 594 (1:200; Invitrogen-Life Technologies). The cells were washed to remove any unbound antibody and then analyzed using a flow cytometer (Millipore).

### Preparation of hydrogel and cell mixture for injection

Hydrogels belong to a class of biological materials and produce a nanoscale environment similar that of the native extracellular matrix [27–30]. A hydrogel consists of 1 % self-assembling peptides (w/v) and 99 % water (w/v). The detailed constituents and properties and the detailed preparation of the hydrogel used in this study are detailed by the manufacturer (Beaver Nano Technologies, China). Briefly, the hydrogel (1 %) was sonicated for 30 min before use. The cells were suspended with 50 µl of 10 % sucrose solution, and 50 µl of the cell suspension was rapidly and carefully mixed with 50 µl of 1 % hydrogel. Then, 100 μl of PBS (Corning) was carefully added to the top of the cell and hydrogel mixture for instantaneous gelation. The 1 % hydrogel (50 µl) diluted with 150 μl of PBS was used as a control.

### Rat renal IR model and injection of RPCs

The renal IR model was generated in 8-week-old male Sprague–Dawley as described previously [16]. Briefly, thirty rats (3–3.5 kg) were anesthetized with chloral hydrate (0.2 mg/g, Sigma), and this step was followed by exposure of the abdomen. For induction of total ischemia in the kidney, the left renal artery was clamped with a small vascular clamp for 45 min. Reperfusion was initiated by removal of the clamp. The right kidney was removed simultaneously. Thirty-four rats survived the IR surgery and no rats died in the following experiments. Then, the hydrogel (200 μl; denoted “IR-control” group) or the suspension of iPSC-derived RPCs (1 × 10^5^ cells in 200 μl of 0.25 % hydrogel per rat; denoted “IR-transplantation” group) was respectively injected into the renal parenchyma of rats (seventeen rats per group). The rats were injected with the immunosuppressant cyclosporine A (Sigma) at a dose of 5 mg/kg per day. Six sham-operated animals underwent similar operative procedures but without clamping of the left renal artery and right kidney nephrectomy. At the indicated time, the blood was collected to measure the levels of blood urea nitrogen (BUN) and serum creatinine (Scr). The rats were killed at the indicated days for the collection of kidney tissue samples. All care and handling of animals was performed with the approval of the Ethical Review Board of Shanghai Six People’s Hospital Affiliated to Shanghai Jiaotong University. The animals were raised in a specific pathogen-free (SPF) environment.

### Histological analysis and tubular injury score

For the histological analysis, the kidney tissues were fixed with 4 % paraformaldehyde overnight and embedded in paraffin. The paraffin sections (4 μm) were stained with hematoxylin and eosin (H&E, Sigma). The quantitative analysis of the renal tubular injury used the grading scores proposed in a previous study [22]. The sections were evaluated by assessing 10 randomly selected high-power fields (40 × objective), and higher scores represented more severe damage: tubular epithelial cell flattening (1 point), brush border loss (1 point), cell membrane bleb formation (1 or 2 points), interstitial edema (1 point), cytoplasmic vacuolization (1 point), cell necrosis (1 or 2 points), and tubular lumen obstruction (1 or 2 points).

### Immunohistochemistry

Immunohistochemical staining was performed with mouse anti-GFP antibody (1:100; Cell Signaling Technology) and mouse anti-proliferating cell nuclear antigen (PCNA, 1:200; Abcam). Briefly, paraffin sections of the kidneys were deparaffinized with xylene and rehydrated in an alcohol series and water. The kidney sections were subjected to antigen retrieval and blocked with a peroxidase-blocking reagent. The sections were incubated with the primary antibody overnight at 4 °C. After washing, the kidney sections were incubated with horseradish peroxidase-conjugated secondary antibody (ZSJQ-BIO, China) for 1 h at room temperature. The sections were visualized with 3,3′-diaminobenzidine (DAB, ZSGQ-BIO) and counterstained with hematoxylin. The number of positive cells was evaluated by counting the stained cells per high-power field in at least 20 randomly selected fields. The apoptotic cells in the kidney were detected by terminal deoxynucleotidyl transferase (TdT)-mediated digoxigenin deoxyuridine nick-end labeling (TUNEL, Promega) staining according to the protocol delineated by the manufacturer. The TUNEL-negative controls were stained without TdT enzyme.

### Statistical analysis

All of the experiments were performed at least three times. The data are shown as the mean ± SEM. Differences were analyzed using one-way analysis of variance (ANOVA). Values of *P* < 0.05 were considered statistically significant.

## Additional file

Additional file 1:
**Figure S1.** Schematic representation and representative bright-field image during the differentiation of mouse iPSCs towards renal progenitor cells. EB, embryoid body. RAB, RA, activin-A and BMP7 in the EB medium. REGM, Renal Epithelial Cell Growth Medium. Scale bar: 100 μm.
