# Supplementary material for: Transplantation of induced pluripotent stem cell-derived renal stem cells improved acute kidney injury
Source: Cell Biosci. 2015 Aug 20;5:45. doi: 10.1186/s13578-015-0040-z (PMC4541730; doi:10.1186/s13578-015-0040-z)
Supplement: Additional file 1: — Figure S1. Schematic representation and representative bright-field image during the differentiation of mouse iPSCs towards renal progenitor cells. EB, embryoid body. RAB, RA, activin-A and BMP7 in the EB medium. REGM, Renal Epithelial Cell Growth Medium. Scale bar: 100 μm. [file 13578_2015_40_MOESM1_ESM.docx]

Additional file

Additional file 1: Figure s1


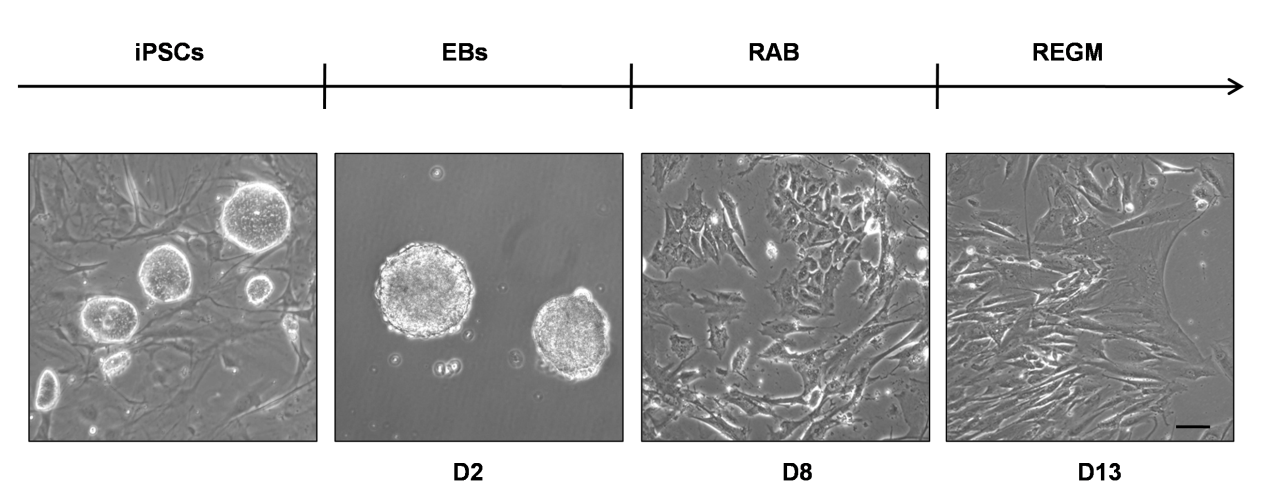


Figure s1. Schematic representation and representative bright-field image during the differentiation of mouse iPSCs towards renal progenitor cells. EB, embryoid body. RAB, RA, activin-A and BMP7 in the EB medium. REGM, Renal Epithelial Cell Growth Medium. Scale bar: 100μm.
